# Zinc Oxide Nanoparticles: A Review on Its Applications in Dentistry

**DOI:** 10.3389/fbioe.2022.917990

**Published:** 2022-05-19

**Authors:** C Pushpalatha, Jithya Suresh, VS Gayathri, SV Sowmya, Dominic Augustine, Ahmed Alamoudi, Bassam Zidane, Nassreen Hassan Mohammad Albar, Shankargouda Patil

**Affiliations:** ^1^ Department of Pedodontics and Preventive Dentistry, Faculty of Dental Sciences, M.S. Ramaiah University of Applied Sciences, Bangalore, India; ^2^ Department of Oral Pathology & Microbiology, Faculty of Dental Sciences, M.S. Ramaiah University of Applied Sciences, Bangalore, India; ^3^ Oral Biology Department, Faculty of Dentistry, King Abdulaziz University, Jeddah, Saudi Arabia; ^4^ Restorative Dentistry Department, Faculty of Dentistry, King Abdulaziz University, Jeddah, Saudi Arabia; ^5^ Department of Restorative Dental Science, College of Dentistry, Shwajra Campus, Jazan University, Jazan, Saudi Arabia; ^6^ Department of Maxillofacial Surgery and Diagnostic Sciences, Division of Oral Pathology, College of Dentistry, Shwajra Campus, Jazan University, Jazan, Saudi Arabia

**Keywords:** zinc oxide nanoparticles, biomedical application, nanodentistry, dental applications, restorative material

## Abstract

Nanotechnology in modern material science is a research hot spot due to its ability to provide novel applications in the field of dentistry. Zinc Oxide Nanoparticles (ZnO NPs) are metal oxide nanoparticles that open new opportunities for biomedical applications that range from diagnosis to treatment. The domains of these nanoparticles are wide and diverse and include the effects brought about due to the anti-microbial, regenerative, and mechanical properties. The applications include enhancing the anti-bacterial properties of existing restorative materials, as an anti-sensitivity agent in toothpastes, as an anti-microbial and anti-fungal agent against pathogenic oral microflora, as a dental implant coating, to improve the anti-fungal effect of denture bases in rehabilitative dentistry, remineralizing cervical dentinal lesions, increasing the stability of local drug delivery agents and other applications.

## 1 Introduction

Nanotechnology, wherein matter is manipulated on a molecular scale, has revolutionized modern dentistry. “Nanodentistry” is the amalgamation of nanotechnology and dentistry and provides the scope for the formulation of innovative materials that can have many potential applications in clinical practice. The nano size confers a larger surface area, allows the controlled synthesis and is also capable of altering the desired physical and chemical properties that enables them for unique interactions with biomolecules. They also have a higher percentage of surface atoms, which maximized their ability due to an increase in surface reactivity (Rasmussen et al., 2010).

Zinc is an essential trace element which is found in the muscle, bone, skin and also in the hard tissues of the tooth. Zinc Oxide Nanoparticle (ZnO NP) is a white colored odorless powder and has a molecular weight of 81.38 g/mol. FDA considers it as a generally recognized as safe (GRAS) substance. Its extensive applications in dentistry are credited to the unique optical, magnetic, morphological, electrical, catalytic, mechanical, and photochemical properties which can be easily altered as per the requirements: by modifying the size, doping with supplementary compounds, or adjusting the conditions of synthesis. As the size of the particles decrease, the desirable characteristics improve (Baek et al., 2012).

In the present, ZnO NPs are being investigated as associates of anti-microbial agents which are one of the most important reasons for its use. A recent theory that explains this is the “Trojan Horse effect”, which states that the acidic lysosomal environment promotes nanoparticle degradation, that in turn brings about conversion of core metals to ions and the release of substances that are toxic and in turn interrupt cell reproduction. Other mechanisms of their anti-microbial action are by locally changing the microenvironments near the microbes and by producing reactive oxygen species (ROS) or by increasing solubility of these nanoparticles. This can induce interplay with -SH group of the enzymes in the microbes and cause malfunction of organelles causing denaturation of the proteins and resulting in damage to DNA. This in turn alters the DNA replication of the microorganisms. Another possible anti-microbial mechanismis by the release of H_2_O_2_ (Şuhani et al., 2018) and by the displacement of Magnesium ions which interferes with the metabolism of the bacteria. The enhanced effect against microbes is attributed to the increased ratio of surface/volume. Hence, the incorporation of ZnO NPs in dental restorative materials, luting materials, tissue conditioners, intracanal medicaments, irrigants, adhesives and other materials can have beneficial anti-microbial effects.

Further research is also being done on this nanoparticle, due to the unlimited fields of application such as regarding its anti-inflammatory activity in response to pathogens, its anti-demineralizing and remineralizing effect on the hard tissues of the tooth, its potential as an anti-cancer agent and many others (Carrouel et al., 2020; Wiesmann et al., 2020). ZnO NPs hence have widespread applications in the field of restorative dentistry, endodontics, regenerative endodontics, prosthetic dentistry, orthodontics, preventive dentistry, implantology and periodontology (Moradpoor et al., 2021). Although ZnO NPs are considered to be a biologically safe material that does not exhibit cell toxicity, however, further research into the regulatory and safety concerns in oral care products on long term use must be discussed, questioned and further researched upon. Majority of the research regarding these NPs are limited to *in-vitro* studies and few animal studies. Therefore, further investigations and clinical trials must be carried out in order to utilize it to its full potential.

## 2 Applications of Zinc Oxide Nanoparticles in Dentistry

**FIGURE 1:**
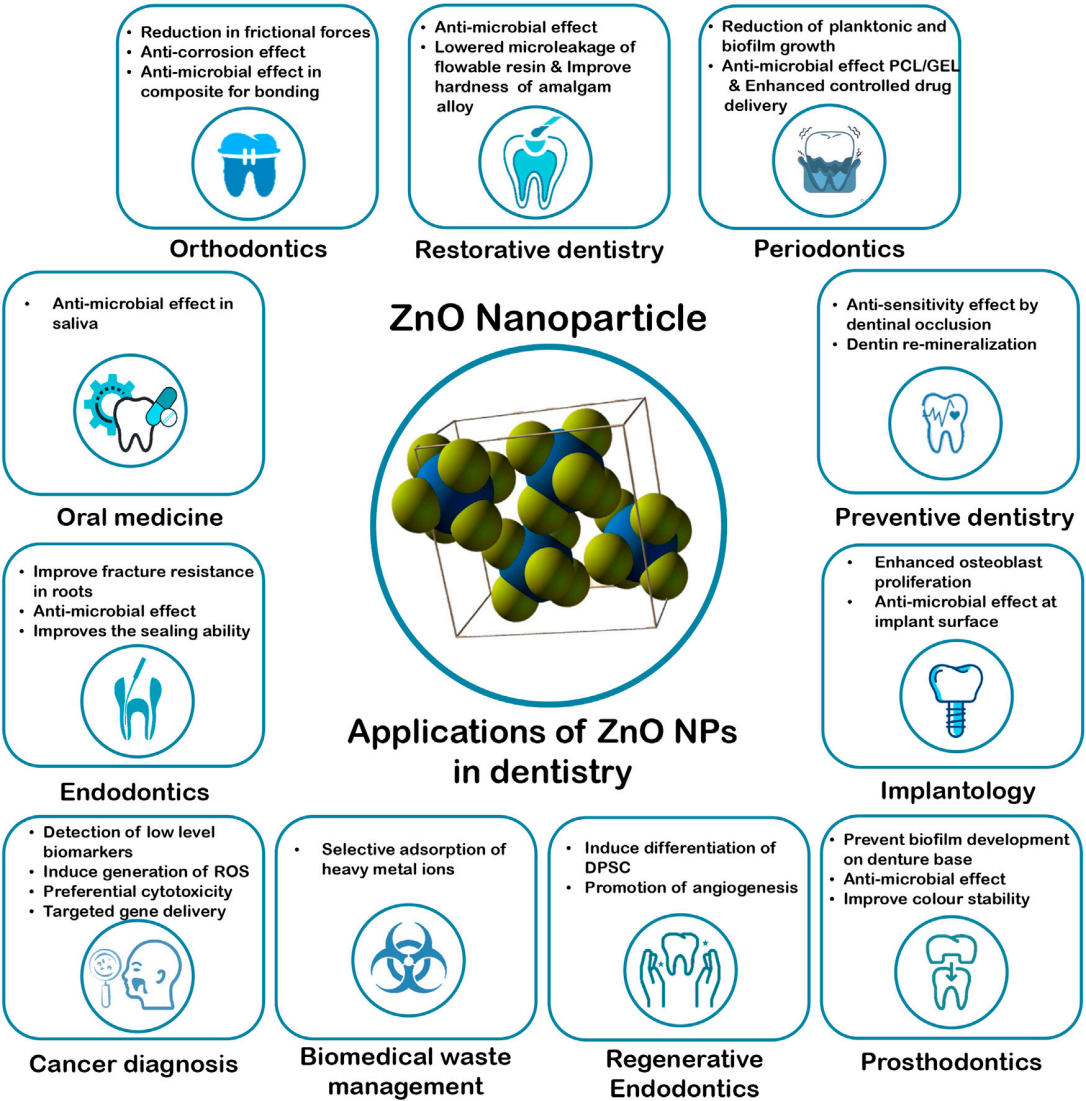
Applications of ZnO NPs in Dentistry.

Zinc Oxide Nanoparticles have a wide range of applications in the various branches of dentistry, such as in the field of restorative dentistry, endodontics, regenerative endodontics, periodontics, prosthodontics, orthodontics, oral medicine, cancer diagnosis, dental implantology, preventive dentistry and biomedical waste management. The research performed using these nanoparticles are summarized in Table1 and Figure 1.

**TABLE 1:** Studies focussing on the Applications of ZnO NPs in dentistry.

Sl No.	Modification	Effect	Author
1	Dental resin composite containing ZnO NP	Inhibition of adhesion of *S. mutans*	Wang et al. (2019)
2	Flowable resin composite with ZnO NP	Decreased microleakage	Teymoornezhad et al. (2016)
3	Flowable resin composite with ZnO NP	Decreased microleakage	Hojati et al. (2013)
4	ZnO NP + resin-based dental composites	Anti-bacterial activity against *S. sobrinus*	Aydin Sevinç and Hanley, 2010)
5	ZnO NP	Anti-bacterial activity against *S. mutans* and *Lactobacillus*	Kasraei et al. (2014)
6	ZnO NP and GIC	Anti-bacterial activity against *S. mutans*	Vanajassun et al. (2014)
7	ZnO NP added into dental adhesive systems	Improved anti-microbial properties	Saffarpour et al. (2016)
8	ZnO NP added into dental adhesive systems	Improved anti-microbial properties	Jowkar et al. (2018)
9	ZnO NP added into dental adhesive systems	Improved anti-microbial properties	Gutiérrez et al. (2019)
10	ZnO NP used as interim cements	Anti-bacterial activity against *S. mutans*	Andrade et al. (2018)
11	ZnO NP	Anti-bacterial activity against *E. coli*	Liu et al. (2009)
12	Combination of ZnO NP and EDTA solution as irrigant	Enhanced fracture resistance of roots	Jowkar et al. (2020)
13	Addition of ZnO NP, calcium hydroxide NPs and chlorhexidine as intracanal medicament	Anti-bacterial activity against *E. faecalis*	Aguiar et al. (2015)
14	ZnO NP as sealer	Enhanced sealing with remineralization of radicular dentin	Toledano et al. (2020)
15	ZnO NP as nanosealer	Reduction in apical microleakage	Javidi et al. (2014)
16	ZnO NP	Improved penetration depth into dentinal tubules	Elkateb et al. (2015)
17	ZnONP added into gutta percha cones	Enhanced anti-bacterial activity against *S. aureus* and *E. faecalis,* excellent hermetic seal	Alves et al. (2018)
18	ZnO NP in Zinc-Bioglass	Induce differentiation of hDPSCs	Huang et al. (2017)
19	ZnO NP in Zinc-Bioglass and Calcium Phosphate Cement	Promotion of odontogenic differentiation and angiogenesis	Zhang et al. (2015)
20	ZnO NP containing composite membranes of PCL/GEL	Anti-bacterial activity against *S. aureus*	ZnO NP + composite membranes of PCL/GEL
21	ZnO NP containingelectrospun membranes of PCL/GEL	Anti-bacterial activity against *P. gingivalis* and *F. nucleatum*	ZnO NP + electrospun membranes of PCL/GEL
22	ZnO NP and serum albumin microspheres with minocycline	Enhanced anti-microbial spectrum, pH-responsiveness, sustained release, tissue-repairing and adhesion and enhanced controlled drug delivery	ZnO NP + serum albumin microspheres containing minocycline
23	ZnO NP added into PMMA in denture bases	Anti-fungal effect against *C. albicans*	Cierech et al. (2019)
24	ZnO NP incorporated into Auto-polymerized acrylic resins	Improvement of flexural strength	Kati. (2019)
25	ZnO NP added into Tissue conditioner	Anti-fungal effect	Homsiang et al. (2020)
26	Combination of ZnONP, chitosan and Ag NP	Anti-microbial effect against *C. albicans, S. mutans, P. aeruginosa* and *E. faecalis*	Mousavi et al. (2020)
27	ZnO NP in silicone prosthesis	Improved colour stability	Charoenkijkajorn and Sanohkan. (2020)
28	ZnO NP coating in NiTi wires	Reduction in the frictional forces, enhanced corrosion resistance and anti-bacterial effect against *S. mutans*	Kachoei et al. (2016)
29	ZnO NP and chitosan NPs in resin-based dental composite bonding agents	Anti-bacterial activity against *S. mutans, S. sanguis and L. acidophilus*	Mirhashemi et al. (2013
30	ZnO NP	Anti-fungal effect against *C. albicans* (oral thrush)	Abd et al. (2015)
31	ZnO NPs and Ag NPs	Enhanced anti-bacterial effect in human and artificial saliva	Pokrowiecki et al. (2019)
32	ZnO NPs	Anti-fungal effect on *Aspergillus niger* and *C. albicans*	Pillai et al. (2020)
33	ZnO NPs	Enhancedsensitivity to *E. coli*, *P. aeruginosa* and Methicillin Resistant *S. aureus*	Ali et al. (2016)
34	Combination of ZnO NPs, Quercetin, Ceftriaxone, Ampicillin, Naringin and Amphotericin B	Enhanced drug efficacy against Methicillin resistant *S. aureus, S. pneumoniae and S. pyogenes, E. coli, S. marcescens* and *P. aeruginosa*	Akbar et al. (2021)
35	ZnO NPs	Detect low-level expression of biomarkers, preferential cytotoxicity against cancer cells, induce ROS.	Rasmussen et al. (2010)
36	ZnO NPs and PEG	Enhanced anti-bacterial activity against *E. coleus* and *S. aureus,* low concentration cytotoxic action against cancer cells	Nair et al. (2008)
37	ZnO NPs and daunorubicin for UV irradiation	Synergistic cytotoxic effects on leukemic cells	Guo et al. (2008)
38	ZnO NPs	Dental wastewater purification treatment	Gu et al. (2020)
39	ZnO NPs	Purification of wastewater	Spoială et al. (2021)
40	ZnO NPs on implant surface	Enhanced proliferation of osteoblasts, anti-bacterial effect against *S. aureus*	Memarzadeh et al. (2015)
41	ZnO NPs on implant surface	Anti-bacterial effect against *S. mutans*	Abdulkareem et al. (2015)
42	ZnO NPs on implant surface	Anti-bacterial effect against *S. mutans*	Kulshrestha et al. (2014)
43	ZnO NPs incorporated into dentifrice	Inhibition of dentin demineralization and enhanced anti-microbial effects	Khan et al. (2020)
44	ZnO NPs nanogels	Promote dentin mineralization	Toledano et al. (2019)
45	ZnO NPs	Dentinal tubule occlusion	Toledano-Osorio et al. (2018)

### 2.1 Restorative Dentistry

ZnO NPs have been found to improve the mechanical and anti-bacterial properties of dental restorative materials. According to a study by Wang et al., it was reported that when ZnO NPs were incorporated in dental resin composites, there was inhibition in the growth and adhesion of *S. mutans*, and in small amounts did not affect the mechanical properties. This is extremely beneficial in not only in the prevention of secondary caries but also in the interception of bulk fracture of the material (Wang et al., 2019). Similarly, in a study done by Teymoornezhad et al., it was reported that incorporation of 3% ZnO NPs on flowable resin composite lowered the microleakage (Teymoornezhad et al., 2016). A comparable outcome was reported in the study by Hojati et al. (2013), on flowable resin composite (Tavassoli Hojati et al., 2013). When 10% ZnO NPs was added to resin-based dental composites, it showed anti-bacterial effectiveness against *S. Sobrinus* (Aydin Sevinç and Hanley 2010). These NPs were also found to exhibit anti-bacterial activity against *S. mutans* and *Lactobacillus* (Kasraei et al., 2014). ZnO NPs when incorporated in Glass Ionomer Cement (GIC) was also found to significantly improve the anti-bacterial properties against *S. mutans* without altering the mechanical properties (Vanajassun et al., 2014).

In various studies, it has been reported that incorporation of ZnO NPs into dental adhesive systems significantly improved the anti-microbial properties without affecting the bond strength adversely (Saffarpour et al., 2016; Jowkar et al., 2018; Gutiérrez et al., 2019). Zinc particles have proven to produce a strong bond at the interface of the dentin and resin by bringing about a decrease in the degeneration of collagen. The hardness of amalgam alloy was found to increase in proportion with the percentage loading of ZnO NPs (Yahya et al., 2013).

ZnO NPs when added to interim cements also exhibited anti-bacterial activity against *S. mutans* (Andrade et al., 2018). ZnO NPs were found to alter the lipid and protein contents of the cell membranes of *E. coli*, which caused distortion leading to leakage of cellular components, ultimately resulting in death (Liu et al., 2009). These properties are extremely beneficial in preventing the occurrence of secondary caries.

However, it was reported that the addition of 1% and 2% by weight of ZnO NPs into GIC did not exhibit anti-microbial activity against strains of *S. mutans*. This might be attributed to the inherent anti-bacterial property of the cement (Garcia et al., 2017). The incorporation of nano-spherical and nano-flower ZnO NPs to GIC was found to decrease the surface hardness, without affecting the flexural strength while incorporation of nano-rod ZnO NPs had no effect on the mechanical properties (Panahandeh et al., 2018). In another study done by Wang et al., it was reported that with the increase in the quantity of ZnO NPs, there was a decrease in the mechanical properties of dental composite resins, with the exception of flexural strength, which may be attributed to the agglomeration of the nanoparticles (Wang et al., 2019). In a systematic review by Arun et al., on the anti-bacterial properties of composite material incorporated with ZnO NPs, it was concluded that the material is unlikely to present a clinical advantage due to the short lifetime of anti-bacterial properties and the poor results against multi-species biofilms (Arun et al., 2021).

### 2.2 Endodontics

The applications of ZnO NPs in endodontics are diverse. In a study by Jowkar et al., When incorporated in EDTA solution for irrigation, the fracture resistance of the roots was enhanced (Jowkar et al., 2020a). In a study done by Aguiar et al., it was reported that these NPs promoted alkalinization and action against *E. faecalis* when used as an intracanal medicament along with calcium hydroxide NPs and chlorhexidine (Aguiar et al., 2015). ZnO NPs when used as an sealer after endodontic therapy was found to exhibit excellent sealing efficacy along with remineralization of the radicular dentin thereby strengthening the tooth (Toledano et al., 2020). It was also reported that ZnO NPs brought about a reduction in the apical microleakage when used as a nano-sealer in endodontics (Javidi et al., 2014). It also significantly improved the penetration depth into the dentinal tubules (Elkateb et al., 2015). Pristine gutta percha cones that were pre-treated argon plasma treatment and coated with ZnO NPs were found to exhibit antibacterial activity against *S. aureus* and *E. fecalis* which provides an excellent hermetic seal thereby reducing chances of reinfection and subsequent endodontic failure (Alves et al., 2018).

However, a study done by Jowkar et al., showed that push-out bond strength of the fiber posts did not improve on the addition of ZnO NPs (Jowkar et al., 2020b). When incorporated into Portland cement (PC) along with ZrO_2_, it was found not to impede with the anti-biofilm activity and to provide radiopacity to the cement. Also, the presence of ZnO NPs significantly reduced the compressive strength of the material (Guerreiro-Tanomaru et al., 2014).

### 2.3 Regenerative Endodontics

Incorporation of these NPs along with SiO_2_, Na_2_O, CaO and P_2_O_5_ to formulate Zinc-Bioglass, was reported to induce the differentiation of human Dental Pulp Stem Cells (hDPSCs) by bringing about an increase in the ALP activity (Huang et al., 2017). Similarly, it was reported that Zinc-Bioglass when incorporated with Calcium Phosphate Cement brought about odontogenic differentiation and also promoted angiogenesis by activating the Wnt, integrin, NF-kB, and MAPK pathways (Zhang et al., 2015). These play a pivotal role in the regeneration of the dentin-pulp tissues.

### 2.4 Periodontics

In the field of periodontal regeneration using guided tissue regeneration, the loading of ZnO NPs into composite membranes of polycaprolactone (PCL) and gelatin (GEL) which were electrospun, brought about reduction in the planktonic and the biofilm growth of the *S. aureus* significantly. These local anti-bacterial properties brought about enhancement in the clinical prognosis of treatments (Prado-Prone et al., 2020). Similarly, when ZnO NPs were incorporated in electrospun membranes made of PCL and PCL/GEL, it showed anti-bacterial activity against *P. gingivalis* and *F. nucleatum* species which in turn brought about an enhanced and better predictable periodontal regeneration (Münchow et al., 2015). ZnO NPs and serum albumin microspheres containing minocycline when incorporated in a Carbopol hydrogel exhibited enhancement of properties such as the anti-microbial spectrum, pH-responsiveness, sustained release, tissue-repairing and adhesion, and also enhanced controlled drug delivery that can increase stability of the drug (Mou et al., 2019).

### 2.5 Prosthodontics

The incorporation of ZnO NPs into the PMMA in denture bases was found to prevent biofilm development by *C. albicans* without exerting a cytotoxic effect on the host cells. Further research can advocate its application as a novel denture base material (Cierech et al., 2019). ZnO NPs in concentrations of 1wt% and 2wt% when incorporated in auto-polymerized acrylic resins was found to improve the flexural strength significantly (Kati 2019). In a study wherein 15 wt% ZnO NPs were incorporated into the tissue conditioner was also found to exhibit an anti-fungal effect (Homsiang et al., 2020). In another study, it was assessed that ZnO NPs along with chitosan and Silver NPs in the concentration of 2.5% inhibited the growth of *C. albicans*, and at a concentration of 5% inhibited the growth of *S. mutans, P. aeruginosa* and *E. faecalis* (Mousavi et al., 2020). The incorporation of 1.5% of ZnO NPs was found to improve the colour stability of silicone prosthesis (Charoenkijkajorn and Sanohkan 2020).

### 2.6 Orthodontics

Nanoparticles have been used in orthodontics to improve the quality of orthodontic treatment either in the form of nano-coated archwires, orthodontic adhesives, and orthodontic brackets (Tahmasbi et al., 2019; Moradpoor et al., 2021). The zinc oxide nanoparticles coated orthodontic appliances minimise bacterial adhesion and enamel demineralization due to its antimicrobial and remineralization potential. Even attempts are made to add ZNO NPs into both orthodontic attachments and bonding materials since they provide a platform for bacterial attachment (Jatania and Shivalinga 2014; Riad et al., 2015; Reddy et al., 2016; Tahmasbi et al., 2019).

It was reported that coating of the NiTi wires with ZnO NPs brought about reduction in the frictional forces by 21% and exhibited anti-bacterial activity against *S. mutans*. It was also reported that ZnO NPs exhibited anti-corrosion effect that enhanced the corrosion resistance propertiesin the orthodontic wires (Kachoei et al., 2016). When a mixture of 10% weight each of ZnO NPs and chitosan NPs was incorporated into are resin-based dental composite bonding agents for the placement of brackets, it exhibited anti-bacterial activity against *S. mutans, S. sanguis* and *L. acidophilus*. This can significantly bring about reduction in the incidence of white-spot lesions during orthodontic therapy (Mirhashemi et al., 2013). Another study investigated that ZNO and CuO NPs coated orthodontic brackets showed better antibacterial activity against *S. mutans*, thus reducing the incidence of dental caries (Ramazanzadeh et al., 2015). It has been reported that when both orthodontic wires and brackets were coated with ZnO NPs the antibacterial potential against *S. mutans* was enhanced and reduced the frictional forces of coated wires (Behroozian et al., 2016). Similarly the stainless steel wires and orthodontic brackets coated with chitosan NPs or ZnO NPs reduced the friction between orthodontic brackets and a Stainless steel wire thus enhances the anchorage control and root resorption risk (Elhelbawy and Ellaithy 2021). Europium ions doped ZnO NPs were incorporated has orthodontic nanoadhesive enhanced the visibility of material for thorough removal of orthodontic adhesive after completion of treatment (Yamagata et al., 2012). It has been reported that orthodontic adhesive with less titanium dioxide, zinc oxide, and silver NPs causes bracket failure because the combination reduces shear bond strength (Reddy et al., 2016). The addition of ZnO to a light cure resin modified GIC as an orthodontic bonding agent improved the original compound’s antimicrobial, physical, and flexural properties (Nuri Sari et al., 2015). Hence ZnO NPs have the potential to be widely used in orthodontic applications to improve treatment outcomes, including increased strength of materials and reduced bacterial count around the orthodontic appliance.

### 2.7 Oral Medicine

ZnO NPs have an inhibitory effect on *C. albicans* in saliva and hence can be used in the treatment of oral thrush, starting from a concentration of 0.05 mg/ml. It was also reported that ZnO NPs along with Silver NPs exhibited enhanced anti-bacterial effect in human and artificial saliva, which can have widespread applications in clinical scenarios (Pokrowiecki et al., 2019). ZnO NPs which are biosynthesized from *Beta vulgaris* was found to exhibit anti-fungal effect on pathogens such as *Aspergillus niger* and *C. albicans* (Pillai et al., 2020). ZnO NPs that are synthesized from Aloe vera leaf extract have been demonstrated to exhibit pronounced sensitivity to *E. coli*, *P. aeruginosa* and Methicillin Resistant *S. aureus,* and hence can be considered as a promising candidate for nano-antibiotics, which deals with the enhancement of the effect against the bacterial strains that are resilient to conventional antibiotics (Ali et al., 2016). These NPs when conjugated with drugs such as Quercetin, Ceftriaxone, Ampicillin, Naringin and Amphotericin B showed enhanced drug efficacy against Methicillin resistant *S. aureus, S. pneumoniae, S. pyogenes, E. coli, Serratia marcescens* and *P. aeruginosa.* Hence, they provide a propitious approach in the combat against disease resistant pathogens (Akbar et al., 2021).

### 2.8 Cancer Diagnosis

ZnO NPs can be implicated in the diagnosis of cancers as it is proven to detect low-level expression of biomarkers which are used for early cancer detection. *In vitro*, it exhibits an inherent preferential cytotoxicity against cancer cells. It also possesses the ability to induce the generation of Reactive Oxygen Species (ROS) due to its semiconductor properties, as the electrons within the NPs can react with O_2_ or hydroxyl ions or water after migrating to the surface to form superoxide and hydroxyl radicals (Manthe et al., 2010). These can set about cell death when the anti-oxidative capacity of the cell is exceeded. Research is being carried out on the utilization of ZnO NPs for gene silencing and targeted gene delivery, which can be utilized to combat cancer (Rasmussen et al., 2010). ZnO NPs that were coated with polyethylene glycol (PEG) were found to exhibit enhanced anti-bacterial activity against *E. coleus* and *S. aureus* by bringing about damage to the cell membrane. They were also found to exhibit a low concentration threshold for cytotoxic action, with a which is due to the upregulation of the Fas ligand on the cell membrane which brings about apoptosis of the cancer cells (Nair et al., 2008). ZnO NPs also exhibits an efficient role in non-surgical tumor ablation method used in cancer therapy. It was demonstrated that ZNO NPs when combined with anti-cancer drug daunorubicin, along with Ultra Violet irradiation, exhibited synergistic cytotoxic effects on the leukemic cells (Guo et al., 2008).

### 2.9 Biomedical Waste Management

The ZnO NPs are found to selectively remove heavy metal ions such as Chromium by adsorption by virtue of their hydroxyl ions. It can therefore be used in dental waste water purification treatment as a green pollutant-diminishing strategy (Gu et al., 2020). Other studies have proven the efficacy of ZnO NPs which can be used in nano-composite membranes used for the purification of water. This is due to its antibacterial activity against *S. aureus* and the favourable photocatalytic activity, which enhances the adsorption of organic pollutants, pesticides and microbes that are found in the wastewaterrendering it safe (Spoială et al., 2021).

### 2.10 Implantology

Chemical modifications of dental implant surfaces with ZNO NPs, which are effective antimicrobial agents, have been carried out in order to reduce the risk of dental implant failure and improve osteointegration. On coating the implant surface with ZnO NPs, the underlying osteoblast cells exhibited an enhanced proliferation after 5 and 10 days. They also exhibited anti-microbial properties against *S. aureus*. These properties are useful to promote bone growth and in the inhibition of infection at the implant site (Memarzadeh et al., 2015). Similar results were reported in studies by Abdulkareem et al. and Kulshrestha et al., on the effect of ZnO NPs against *S. mutans* biofilm on dental implant surfaces (Kulshrestha et al., 2014; Abdulkareem et al., 2015). According to the findings, ZNO bio-functionalized thin films containing DMP1 peptides can improve the physicochemical, osteogenic, apatite nucleation and corrosion resistance properties of this material suggesting promising applications in dental implant (Trino et al., 2018).

Titania (Ti)-zinc (Zn)-oxide nanocomposite-(nC) thin films were co-sputtered to strengthen the cohesiveness of metallic fixtures with bone. The developed thin film also exhibited strong antibacterial activity against *S. aureus* and *E. coli* (Goel et al., 2019). Modified titanium implant materials developed using N-halamine and ZnO nanoparticles demonstrated remarkable antibacterial activity against *P. aeruginosa*, *E. coli*, and *S. aureus* without using antibiotics (Li et al., 2017). Titanium implants with coatings of Poly (lactic-co-glycolic acid)/Silver/ZnO nanorods demonstrated long-lasting antibacterial activity against *S. aureus* and *E. coli*, as well as excellent cytocompatibility and biocompatibility (Xiang et al., 2017).

### 2.11 Preventive Dentistry

ZnO NPs incorporated in a dentifrice was found to cause dentinal tubule occlusion. These can also be incorporated as preservatives in dentifrices as it not only brings about inhibition of dentin demineralization but also exhibits enhanced anti-microbial effects (Khan et al., 2020). Its incorporation and in nanogels and application on eroded cervical dentin, was found to promote dentin mineralization (Toledano et al., 2019). These can be utilized in achieving an anti-sensitivity effect. Studies have shown that dentin which is treated with ZnO NPs exhibited greater ability to produce dentinal tubule occlusion which makes it an effective agent in the treatment of dentinal hypersensitivity 67]. The ZnO NPs treated dentin was found to have higher levels of proteoglycans that act as bonding agents between the HAp crystals and collagen network. Further, they enhance the release small integrin-binding ligand N-linked glycoproteins and small leucine-rich proteoglycans from dentin through Matrix Metalloproteinase-3 activity. These proteins take part in the mineralization of dentin, and the immobilized phosphorylated proteins induce formation of mineral. Zinc NPs also reduces the collagen degradation which is mediated by Matrix Metalloproteinase-3 in dentin that is partially demineralized and hence promotes dentin re-mineralization (Toledano-Osorio et al., 2018; Toledano et al., 2019).

## 3 Conclusion

Nano-dentistry has opened a new standpoint for revolution in oral care and portrays a growing field with the capability to address the new and improved applications in dentistry. ZnO NPs have a broad spectrum of applications the various fields of dentistry such as restorative dentistry, endodontics, regenerative endodontics, periodontology, prosthodontics, orthodontics, implantology, preventive dentistry among other fields. The use of ZnO NPs represents a broadening horizon for the diagnosis, treatment, and prevention of various oral conditions, and in enhancing the characteristics of existing dental materials. It is hence crucial to strengthen the symbiosis between cliniciansand materials scientists asnano-dentistry is still technologydriven, with many roadblocks ahead. However, most of the research is still in the development pipeline and for realizing the complete *in vivo* potential in dentistry, further research that focus on its clinical implications should be carried out.
